# Fedratinib, a newly approved treatment for patients with myeloproliferative neoplasm-associated myelofibrosis

**DOI:** 10.1038/s41375-020-0954-2

**Published:** 2020-07-09

**Authors:** Moshe Talpaz, Jean-Jacques Kiladjian

**Affiliations:** 1 University of Michigan Comprehensive Cancer Center, Ann Arbor, MI USA; 2 Hôpital Saint-Louis, Université de Paris, Inserm, Paris, France

**Keywords:** Drug development, Myeloproliferative disease

## Abstract

Myeloproliferative neoplasm (MPN)-associated myelofibrosis (MF) is characterized by cytopenias, marrow fibrosis, constitutional symptoms, extramedullary hematopoiesis, splenomegaly, and shortened survival. Constitutive activation of the janus kinase/signal transducer and activator of transcription (JAK/STAT) signaling pathway in MF leads to cell proliferation, inhibition of cell death, and clonal expansion of myeloproliferative malignant cells. Fedratinib is a selective oral JAK2 inhibitor recently approved in the United States for treatment of adult patients with intermediate-2 or high-risk MF. In mouse models of *JAK2*V617F-driven myeloproliferative disease, fedratinib blocked phosphorylation of STAT5, increased survival, and improved MF-associated disease features, including reduction of white blood cell counts, hematocrit, splenomegaly, and fibrosis. Fedratinib exerts off-target inhibitory activity against bromodomain-containing protein 4 (BRD4); combination JAK/STAT and BRD4 inhibition was shown to synergistically block NF-kB hyperactivation and inflammatory cytokine production, attenuating disease burden and reversing bone marrow fibrosis in animal models of MPNs. In patients, fedratinib is rapidly absorbed and dosed once daily (effective half-life 41 h). Fedratinib showed robust clinical activity in JAK-inhibitor-naïve patients and in patients with MF who were relapsed, refractory, or intolerant to prior ruxolitinib therapy. Fedratinib is effective regardless of JAK2 mutation status. Onset of spleen and symptom responses are typically seen within the first 1–2 months of treatment. The most common adverse events (AEs) with fedratinib are grades 1–2 gastrointestinal events, which are most frequent during early treatment and decrease over time. Treatment discontinuation due to hematologic AEs in clinical trials was uncommon (~3%). Suspected cases of Wernicke’s encephalopathy were reported during fedratinib trials in ~1% of patients; thiamine levels should be monitored before and during fedratinib treatment as medically indicated. Phase III trials are ongoing to assess fedratinib effects on long-term safety, efficacy, and overall survival. The recent approval of fedratinib provides a much-needed addition to the limited therapeutic options available for patients with MF.

## Introduction

Myelofibrosis (MF) is a life-threatening condition characterized by hematopoietic stem-cell-derived clonal proliferation, abnormal cytokine production, bone marrow fibrosis, anemia, splenomegaly, a large array of symptoms, extramedullary hematopoiesis, leukemic progression, and shortened survival [1]. MF can appear de novo (primary MF) or secondary to polycythemia vera (PV) or essential thrombocythemia (ET) [2]. MF symptoms related to splenomegaly (abdominal distension and pain, early satiety, splenic infarction, dyspnea, and diarrhea), constitutional symptoms (fatigue, cachexia, pruritus, bone pain, weight loss, night sweats, and fever) and anemia significantly compromise patients’ quality-of-life [3].

Janus kinase 2 (JAK2) has an essential role in signaling of normal hematopoiesis. The somatic *JAK2*V617F activating mutation is found in 50–60% of patients with primary MF and ET, and in 95% of patients with PV [4]. *JAK2*V617F constitutively activates the JAK/STAT signaling pathway, resulting in cell proliferation and clonal expansion of myeloid malignant cells [5]. Mutations that cause abnormal activation of myeloproliferative leukemia protein (*MPL*, the thrombopoietin receptor) are the founding driver mutations in about 5% of MPNs [6]. Point mutations in *MPL* result in a more activated receptor that also signals through JAK2. Indirectly, frameshift mutations in calreticulin (*CALR*), a protein folding chaperone, lead to expression of a truncated protein with aberrant binding activity that creates a permanent interaction with MPL. This abnormal CALR–MPL complex leads to constitutively active signaling in the absence of thrombopoietin stimulation [7]. These three distinct mechanisms all share the downstream consequences of unregulated thrombopoietin/JAK2 signaling that ultimately leads to the overproduction of megakaryocytes in MPNs. Neither *CALR* nor *MPL* mutations activate the *JAK2* pathway to the same degree that *JAK2*V617F does, which may partially explain the heterogeneity of symptoms and progression among MPN patients [6]. Additional molecular aberrations frequently detected in MF include *ASXL1*, *IDH1/2*, *EZH2*, and *SRSF2*, which may contribute to disease progression and leukemic transformation [1, 8, 9]. These “high molecular risk” mutations are more frequent in MF than in ET and PV, and may in part explain the reduced survival and higher rate of leukemic transformation in MF patients compared with the other MPNs [1, 8, 10].

Treatment decisions for MF are not currently driven by the molecular profile or disease subtype (primary, post-PV, or post-ET MF); rather, they are influenced mainly by symptom burden and MF disease risk category, as determined by validated prognostic scoring systems [5, 11–14]. Symptomatic low-risk MF is often managed with Peg-IFN-α-2a, hydroxyurea, immunomodulatory drugs (e.g., lenalidomide) [15, 16], or ruxolitinib (approved in Europe but not in the USA for symptomatic low-risk MF) [17], and a variety of agents are used to address MF-related anemia (e.g., erythropoiesis stimulating agents [18], danazol [19]), although none of these drugs are specifically approved for this indication. For patients with intermediate- or high-risk MF, bone marrow or hematopoietic stem cell transplant can prolong survival and is potentially curative, but few patients are eligible due to advanced age, frequent comorbidities, or poor performance status [20], and transplant is associated with high rates of morbidity and mortality, particularly in patients with poor prognostic disease features [21, 22]. Continued use of JAK inhibitors in the peritransplant setting is being explored, and early results suggest ruxolitinib could improve transplant outcomes [23]. For patients who are transplant ineligible, treatment with JAK2 inhibitors is shown to reduce MF-associated splenomegaly and symptom burden [24–28].

Until recently, ruxolitinib, a dual JAK1/JAK2 inhibitor that was approved by the United States (US) Food and Drug Administration (FDA) in 2011, was the only available drug indicated for treatment of intermediate- and high-risk MF [29]. Evidence suggests there may be a survival benefit with ruxolitinib compared with conventional therapies [30, 31]. However, many patients treated with ruxolitinib lose response, have a suboptimal response, or develop cytopenias during treatment, resulting in ruxolitinib discontinuation within a few months and subsequent risk of disease rebound [32, 33]. In the phase III COMFORT-I and COMFORT-II trials, pooled ruxolitinib discontinuation rates at 3 and 5 years were ~50% and ~70%, respectively [30, 31]. Suboptimal ruxolitinib dosing to avoid treatment-related adverse events (AEs), at least initially [33], appears to be relatively common [32].

Fedratinib (INREBIC®; formerly TG101348/SAR302503) is an oral, potent JAK2 inhibitor with activity against wild-type and mutationally activated JAK2 and FLT3. In August 2019, the US FDA approved fedratinib for treatment of adult patients with intermediate-2 or high-risk primary or secondary MF [34]. The National Comprehensive Care Network (NCCN) clinical practice guidelines for treatment of MPNs now includes fedratinib as an option for patients with intermediate-2 or high-risk MF with platelet counts ≥50 × 10^9^/L, used as initial therapy or as second-line therapy for patients previously treated with ruxolitinib [16].

The clinical activity of fedratinib in MF, at the recommended starting dose of 400 mg/day has been assessed in JAK-inhibitor-naïve patients and in patients previously treated with ruxolitinib [24, 28, 35]. Here, we present a comprehensive overview of fedratinib pharmacology, clinical development, efficacy, safety, and AE management strategies, and a description of ongoing clinical investigations of fedratinib in patients previously treated with ruxolitinib.

## Pharmacology

### JAK2 selectivity

**Fig. 1:**
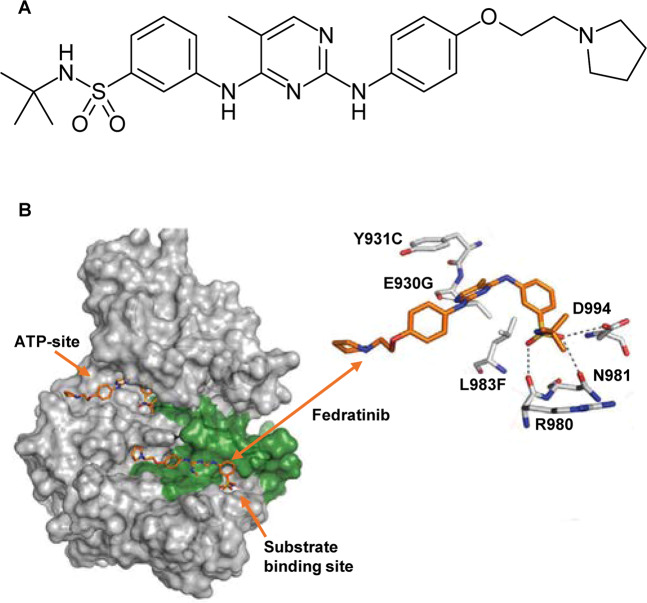
Fedratinib molecule. **a** Fedratinib chemical structure; **b** Dual-binding activity of fedratinib at the JAK2 948 ATP and peptide-substrate binding sites [36].

Fedratinib was synthesized using structure-based drug design to create a JAK2-selective inhibitor with higher potency for JAK2 over closely related kinase family members. Structural modeling showed dual binding of fedratinib in the kinase domain at both the ATP and peptide-substrate binding sites (Fig. 1), which may help explain a lack of genetic resistance to fedratinib [36]. In in vitro studies, 211 ruxolitinib-resistant *JAK2* variants show little or no resistance to fedratinib (mechanisms of ruxolitinib resistance have not yet been identified) [36]. In cell-free kinase activity assays, fedratinib has a IC_50_ value for wild-type *JAK2* and *JAK2*V617F of 3 nM, which is 35 times lower than for *JAK1*, >300 times lower than for *JAK3*, and >100 times lower than for *TYK2* (Table 1) [37].

**Table 1:** Fedratinib is JAK family selective and relatively specific [37].

Kinase selectivity of fedratinib			
Primary target		JAK family kinases	
Kinase	Enzyme IC_50_ (nM)	Kinase	Fold selectivity^a^
JAK2	3	JAK2	1
JAK2V617F	3	JAK3	334
FLT3	15	JAK1	35
RET	48	TYK2	135

No other tested kinase had an IC_50_ < 50 nM.

^a^Fold selectivity compared with JAK2.

### FLT3 inhibition

The JAK/STAT signaling pathway may be hyperactivated even in the absence of known driver mutations, and, like ruxolitinib [26], fedratinib can elicit responses in patients with MF not harboring a *JAK2*, *CALR*, or *MPL* mutation (“triple negative” MF), potentially due to its inhibitory activity against other kinases [24]. Fedratinib exhibits off-target inhibition of mutant and wild-type *FLT3*, a tyrosine kinase expressed on hematopoietic stem cells and myeloid progenitor cells that plays an important role in cell survival and proliferation [38, 39]. Activation of *FLT3* ultimately leads to phosphorylation and activation of the PI3K/AKT, MAPK, and STAT5 signaling pathways involved in multiple anti-apoptotic, proliferation, and differentiation pathways [38, 39]. Signaling via the FLT3 ligand and FLT3-mediated activation of p38-MAPK play a role in the inflammatory dysmegakaryopoiesis characteristic of primary MF, and are associated with disease progression to blast phase [40–42]. *FLT3* and *JAK2* mutations appear to be mutually exclusive in patients with MPNs [43, 44]; whether activity against *FLT3* contributes to the therapeutic effect of fedratinib is unknown.

### BRD4 inhibition

Fedratinib exerts inhibitory activity against BRD4; dual targeting of JAK2 and bromodomains may contribute to the therapeutic efficacy of fedratinib [45–47]. Members of the BET protein family (BRD2, BRD3, BRD4, and BRDT) are implicated in various cancers [48, 49]. Epigenetic bromodomains regulate transcription, chromatin remodeling, gene splicing, protein scaffolding, and signal transduction, and as such, have fundamental roles in cell proliferation and division [48, 50]. Constitutive activation of JAK/STAT in MPN cells leads NF-κB activation and collaboration between JAK/STAT and NF-kB pathways activated by inflammatory stimuli promote aberrant cytokine production and MF progression [51]. The BET family of proteins further augments pro-inflammatory signals [51]. Combination JAK/STAT inhibition and BET inhibition treatment has been shown to synergistically block NF-kB hyperactivation and inflammatory cytokine production, thereby attenuating disease burden and reversing bone marrow fibrosis in mouse models of MPN [47].

A screening assay of 628 kinase inhibitors to determine activity against the first bromodomain of BRD4, which is overexpressed in many cancers [52], showed fedratinib was among nine kinase inhibitor drugs to exert potent bromodomain inhibition with nanomolar activity (IC_50_ value 164 ± 10 nM) at therapeutically relevant concentrations [45]. Additionally, a cross-screening panel of 46 bromodomains outside the BET family showed fedratinib also interacted with bromodomains of histone acetyl transferases. Fedratinib potently suppressed c-Myc expression (a well-established marker of BET inhibition) in MM.1S multiple myeloma cells; in contrast, c-Myc was not suppressed by selective JAK inhibitors that lacked BET activity, including ruxolitinib [45].

## Pharmacokinetics

A randomized, placebo-controlled, phase I study in healthy volunteers was conducted to assess the pharmacokinetics, pharmacodynamics, and tolerability of single fedratinib doses ranging from 10 to 680 mg [53]. Fedratinib was rapidly absorbed, reaching peak plasma concentrations at 2–3 h after initial dosing. Fedratinib exposure appeared to increase in a greater than dose‐proportional manner, with an increase in exposure in the 80–500 mg dose range ~3-fold higher than would be expected with dose proportionality [53]. Similarly, in patients with MF receiving fedratinib doses of 30–800 mg/day, mean steady-state maximum plasma concentration (*C*_max_) and area under the concentration-time curve (AUC_0-t_) values increased ~54- and 88-fold, respectively, over the 27-fold increase in dose [35]. The bioavailability of fedratinib is minimally impacted by food, and it can be taken with or without a meal [54]. Fedratinib has an effective half-life of 41 h [34]. Steady-state fedratinib plasma concentrations were achieved by day 15 of treatment [34, 55]. Fedratinib is metabolized by CYP3A4, CYP2C19, and FMO3; co-administration with strong or moderate CYP3A4 inhibitors (e.g., ketoconazole) may increase fedratinib exposure [34]. After oral administration, fedratinib accounts for approximately 80% of total circulating drug in plasma, and following a single oral dose, 77% of the administered dose (23% unchanged) was excreted in feces and 5% (3% unchanged) was eliminated in urine [34].

No clinically meaningful effect of age, race, sex, body weight, or mild-to-moderate renal or hepatic impairment on PK parameters has been observed with fedratinib [34].

## Pharmacodynamics

### JAK/STAT signaling

In cell models expressing mutationally active *JAK2* or *FLT3*-ITD, fedratinib reduced phosphorylation of downstream STAT3/5 proteins (pSTAT3/pSTAT5), inhibited cell proliferation, and induced apoptotic cell death [37, 53]. Similarly, in mouse models of *JAK2*V617F-driven myeloproliferative disease, fedratinib blocked pSTAT5, increased survival and improved disease-associated features, including reduction of white blood cell (WBC) counts, hematocrit, splenomegaly, and reticulin fibrosis [37]. In patients with MF treated with fedratinib 300, 400, or 500 mg per day in a phase II dose-finding study (NCT01420770), pSTAT3 levels were reduced at all dose levels within 2 h of the first fedratinib dose [55]. Mean reductions in pSTAT3 levels from baseline were 47.9%, 50.3%, and 46.4% in the 300 mg, 400 mg, and 500 mg dose groups, respectively [55]. Similar reductions in pSTAT3 were seen at fedratinib trough levels at steady state on days 15 and 29 of treatment. Patients with greater reductions in pSTAT3 levels were more likely to achieve a spleen response during fedratinib treatment [55].

### *JAK2*V617F

Fedratinib has demonstrated activity against cells expressing *JAK2*V617F in animal models [37, 56, 57]. The effect of fedratinib on *JAK2*V617F allele burden in patients with MF is less clear. In a multicenter, phase I study of fedratinib at doses ranging from 30 to 800 mg daily in 59 patients with intermediate- or high-risk MF, significant decreases in *JAK2*V617F variant allele frequency (VAF) were observed at 6 cycles (*P* = 0.04) and 12 cycles (*P* = 0.01) in *JAK2-*mutated patients (*n* = 51), suggesting direct activity against the malignant clone [35]. In that study, reductions in *JAK2*V617F VAF were more pronounced in patients with higher mutation burden at baseline: following 24 cycles of fedratinib treatment, patients who began the study with a VAF > 20% showed a persistent decrease in the median percentage *JAK2*V617F mutant allele burden compared with baseline (21% vs. 60% at baseline; *P* = 0.03) [58]. In contrast, in a phase II study of 31 patients with MF treated with fedratinib doses of 300, 400, or 500 mg daily, no consistent change in *JAK2*V617F allele burden was observed during the course of treatment [55].

### Cytokine expression

**Fig. 2:**
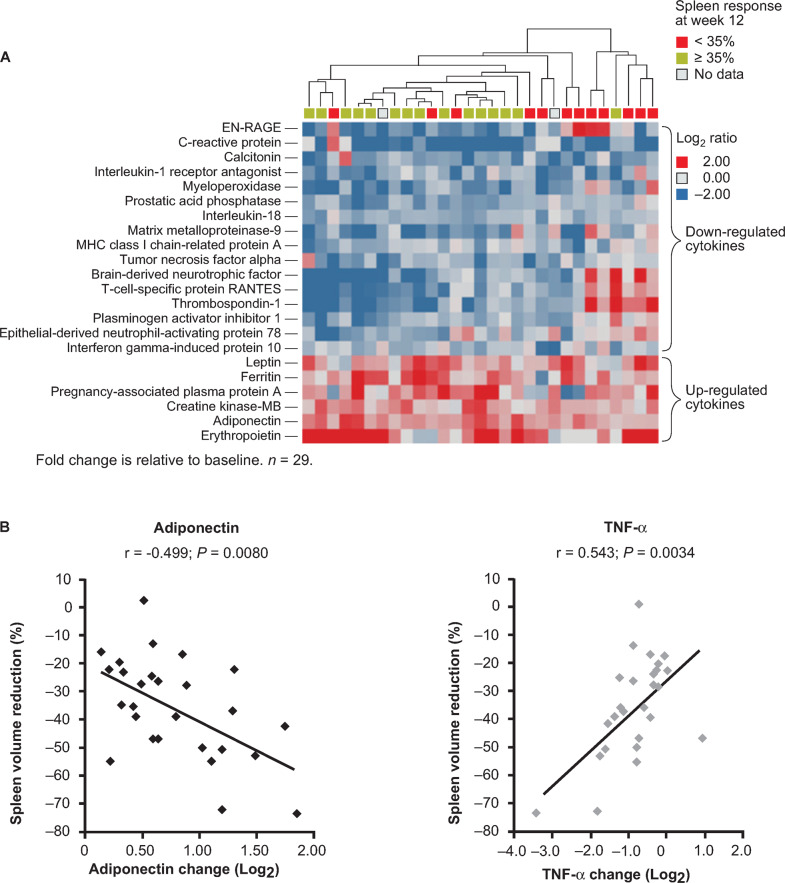
Cytokine regulation by fedratinib. **a** Hierarchical clustering of patients by changes in the 22 regulated cytokines at week 4; **b** Correlation between changes in levels of adiponectin and TNF-α and reduction in spleen volume [55].

Abnormal cytokine expression is thought to contribute to MF-related bone marrow stromal changes, ineffective erythropoiesis, extramedullary hematopoiesis, and constitutional symptoms [59, 60]. In the phase II dose-finding study mentioned above, plasma levels of 28 cytokines (among 97 cytokines screened) were significantly modulated (≥1.5-fold change; *P* < 0.05, adjusted for multiple comparisons) compared with baseline over the first 12 weeks of fedratinib treatment [55]. The highest level of upregulation occurred in erythropoietin, ferritin, adiponectin, and leptin; the greatest downregulation occurred in C-reactive protein, the T-cell-specific protein RANTES (regulated upon activation normal T-cell expressed), and the EN-RAGE (extracellular newly identified receptor for advanced glycation end products) binding protein. Hierarchical clustering of patients according to changes in cytokine expression showed that patients with a spleen response at 12 weeks had similar patterns of cytokine regulation within the first 4 weeks of treatment (Fig. 2a). Of cytokines showing a ≥1.5-fold change at week 12, 8 cytokines correlated with spleen volume reductions met criteria for statistical significance (*P* < 0.05 when adjusted for false discovery rate), including significant downregulation of TNF-α, which can promote expansion of the *JAK2*V617F mutant clone [61], and significant upregulation of adiponectin (Fig. 2b). Adiponectin has anti-inflammatory and anti-fibrotic activity and inhibits the expression of NF-κB target genes, leading to downregulation of both inflammatory cytokines (e.g., TNF-α) and profibrotic TGF-β signaling [62, 63]. Increased production of adiponectin and concurrent decreased expression of inflammatory cytokines may contribute to fedratinib efficacy for reducing spleen volume and improving symptom burden.

Ruxolitinib also suppresses inflammatory cytokine production [64], though fedratinib and ruxolitinib may do so via different mechanisms. Many pro-inflammatory cytokines signal through JAK1-dependent cellular pathways, and the suppressive effect of ruxolitinib on inflammatory cytokines may be linked to its inhibitory activity against JAK1 [64, 65]. JAK1 inhibition also negatively affects T-cell signaling capacity and proliferation [66], factors that may directly contribute to the variety of infectious complications seen with ruxolitinib. Specificity for JAK2 inhibition with fedratinib may reduce the incidence of infectious events due to less immunosuppression [67]. Fedratinib is a weaker inhibitor of JAK1 and downregulation of inflammatory mediators more likely reflects its inhibition of BRD4 and BET proteins, and consequent attenuation of NF-κB hyperactivation [37, 47, 51].

### Anti-fibrotic effects

In the extension phase of a phase I dose-ranging study in patients with MF receiving fedratinib doses of 120–680 mg per day in consecutive 4-week cycles (median daily dose 489.6 mg), bone marrow samples were collected before fedratinib treatment, at the end of treatment cycle 6, and following each additional 6 cycles of therapy [68]. Bone marrow fibrosis had stabilized or improved from baseline in 15/18 evaluable patients by the end of 6 treatment cycles, and in 4/9 patients by cycle 30. Two patients showed complete resolution of bone marrow fibrosis by cycle 12 of fedratinib treatment [68]. These findings require confirmation in blinded review by expert pathologists in larger patient populations. Interestingly, a recent study in an animal model of acute liver injury supports the putative anti-fibrotic effects of fedratinib: in a mouse model of CCl4-induced hepatic injury, fedratinib attenuated collagen accumulation and hepatic stellate cell activation, and reduced inflammatory macrophage infiltration and activation in the liver in vivo, resulting in decreased intrahepatic inflammation and fibrogenesis [69].

## Early dose-finding studies

Clinical use of fedratinib was first evaluated in a phase I dose-escalation study in 59 patients with intermediate or high-risk primary, post-PV, or post -ET MF (TED12037; NCT00631462) [35]. Doses ranged from 30 to 800 mg/day, administered in consecutive 4-week cycles. The lowest fedratinib dose with clinical activity was 240 mg and the maximum tolerated dose was 680 mg/day [35]. The dose-limiting toxicity was an asymptomatic grades 3–4 increase in serum amylase in 2/6 patients that reversed upon treatment cessation. The most common AEs were mainly grade 1 gastrointestinal events; grade 3 nausea, diarrhea, and vomiting were reported for 3%, 10%, and 3% of all patients, respectively, and were dose-dependent, reported almost exclusively at fedratinib doses at or above 680 mg/day. Grades 3–4 treatment-related hematologic AEs included anemia (13/37 non-transfusion-dependent patient at baseline [35%]), thrombocytopenia (24%,) and neutropenia (10%), with most occurring in the first three fedratinib treatment cycles. Of the 13 patients who developed grades 3–4 anemia (all in the 680 mg dosing cohort), two-thirds (67%) had grade 2 anemia at study entry and 9 of 14 patients who developed grades 3–4 thrombocytopenia had grades 1–2 thrombocytopenia at baseline. Serious AEs considered at least possibly related to fedratinib occurred in eight patients, and included asymptomatic hyperlipasemia, thrombocytopenia/neutropenia, depression, tumor lysis syndrome, cerebrovascular accident, and dehydration [35].

Onset of spleen response was generally seen within the first 2 months of fedratinib treatment. Of patients who completed 6 months of fedratinib, 61% achieved a ≥25% reduction in palpable spleen size and 39% had a ≥50% reduction that persisted for ≥8 weeks. Of 28 patients who entered the trial with leukocytosis (WBC count > 11 × 10^9^/L), 16 patients (57%) achieved normal WBC counts after 6 months of fedratinib treatment. Similarly, 9 of 10 patients (90%) who entered the trial with thrombocytosis (platelet count > 450 × 10^9^/L) attained normal platelet counts [35].

This study, which began in 2008, included a long-term open extension phase (TED12015; NCT00724334) that currently provides the longest reported exposure to fedratinib therapy. At a reported follow-up in 2011, 23 of the original 59 patients (39%) remained on fedratinib treatment [58]; the median number of fedratinib cycles received was 30 (range 13–44) and the median current fedratinib dose was 440 mg/day. Proportions of patients with a ≥50% reduction in spleen size were 54% at 6 months (*n* = 57); 67% at 12 months (*n* = 42); 53% at 18 months (*n* = 36); 55% at 24 months (*n* = 31); and 61% at 30 months (*n* = 18) [58].

In a phase II randomized, dose-finding study (NCT01420770), 31 patients with intermediate-2 or high-risk MF received fedratinib 300 mg (*n* = 10), 400 mg (*n* = 10), or 500 mg (*n* = 11) per day in 28-day cycles for up to 48 weeks [55]. The median numbers of fedratinib cycles received were 13.0 (range 2–16), 14.0 (6–16), and 13.0 (7–17) in the 300 mg, 400 mg, and 500 mg dosing arms, respectively. At week 24, mean [±standard deviation] reductions in spleen volume relative to baseline for the intention-to-treat (ITT) population were –26.6% [±14.2%], –37.2% [±22.5%], and –41.1% [±22.0%] in the fedratinib 300 mg, 400 mg, and 500 mg daily dose groups, respectively, and spleen volume response rates (SVRR; the proportion of patients who achieved a ≥35% decrease in spleen volume from baseline as measured by MRI) were 30, 60, and 55% [55]. At week 48, SVRRs were 30% in the fedratinib 300 mg/day arm, 80% in the fedratinib 400 mg/day arm, and 45% in the fedratinib 500 mg/day arm. The study was not powered to detect statistical differences in SVRRs among the three dosing groups. Grade 3 or grade 4 treatment-emergent AEs (TEAEs) were reported for 8/10 patients in the 300 mg dose group, 8/10 in the 400 mg dose group, and for all 11 patients in the 500 mg dose group. The most frequent hematologic abnormality was anemia, which was reported for all 31 patients; grades 3–4 anemia occurred in 60%, 50%, and 64% of patients in the fedratinib 300 mg, 400 mg, and 500 mg/day dosing arms, respectively. In the fedratinib 300 mg and 400 mg dose groups, mean hemoglobin levels reached nadir at ~12–16 weeks from treatment initiation, then tended to increase to baseline or above-baseline levels by week 48. Mean hemoglobin levels were lower in the fedratinib 500 mg/day dosing group during treatment.

Thus, results of early dose-finding studies supported an initial 400 mg daily fedratinib dose as having the optimal risk-benefit profile for treatment of MF [24, 28, 55].

## Efficacy in pivotal trials

### JAKARTA

The pivotal phase III, multicenter, randomized, double-blind, placebo-controlled JAKARTA trial evaluated the safety and efficacy of once-daily fedratinib 400 or 500 mg vs. placebo in 28-day cycles, in JAK-inhibitor-naïve patients with MF. Regulatory approval of the recommended fedratinib 400 mg daily dose for treatment of patients with intermediate-2 or high-risk MF in the United States was based in large part on the clinical efficacy and safety of the 400 mg dose in the JAKARTA trial [24, 34].

Key eligibility criteria included primary or secondary (post-PV or post-ET) MF, intermediate-2 or high-risk disease, palpable splenomegaly, platelet count ≥ 50 × 10^9^/L, and Eastern Cooperative Oncology Group (ECOG) performance status score of ≤2 [24]. The primary end point was SVRR, the proportion of patients who achieved a ≥35% reduction in spleen volume from baseline (confirmed by MRI/CT) at the end of cycle 6 (EOC6), confirmed 4 weeks later. A key secondary endpoint was symptom response rate, defined as the proportion of patients who achieved a ≥50% decrease in total symptom score (TSS) on the modified Myelofibrosis Symptom Assessment Form (MFSAF [70]) from baseline to EOC6. In the placebo arm, patients were treated for 6 months or until disease progression, after which they could crossover to fedratinib treatment.

**Fig. 3:**
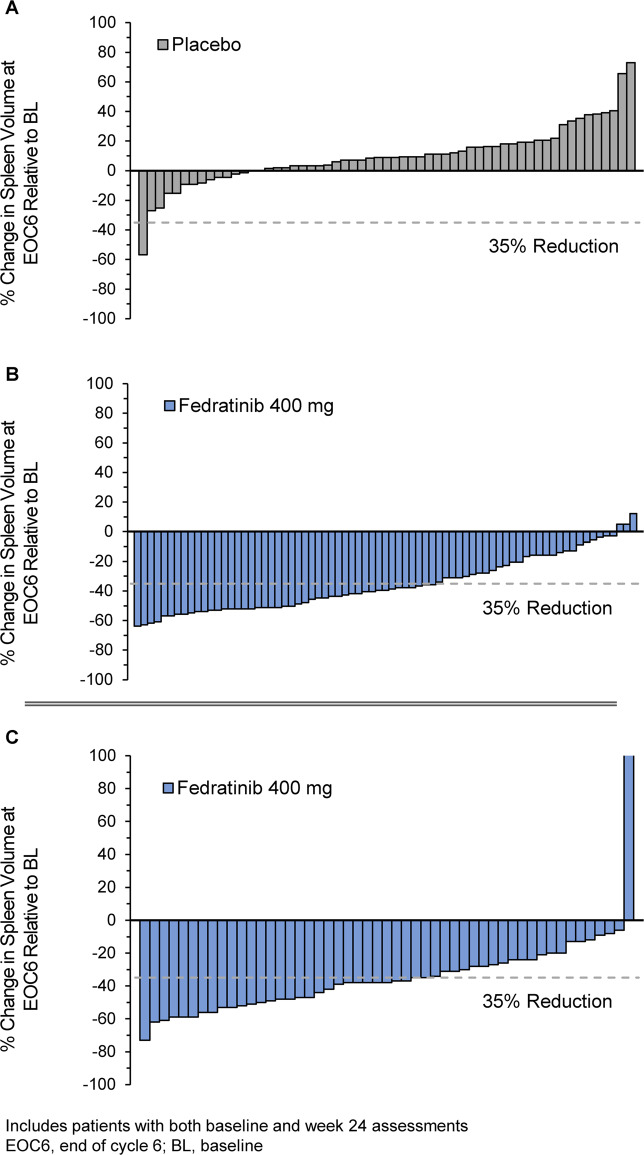
Spleen volume response. Individual spleen volume changes at the end of treatment cycle 6 in JAKARTA with placebo (**a**) and fedratinib 400 mg/day (**b**); [34] and in JAKARTA2 with fedratinib 400 mg/day (**c**) in patients previously treated with ruxolitinib [71].

Of all 289 patients enrolled, a total of 96 patients in JAKARTA were randomized to receive fedratinib 400 mg and 96 patients were randomized to placebo (1 patient did not receive placebo and was not included in safety analyses). Baseline characteristics are shown in Supplementary Table 1. The total median duration of exposure to fedratinib 400 mg daily in the JAKARTA trial was 15.5 months [34]. SVRR at EOC6 with a follow-up scan 4 weeks later was 37% in the fedratinib 400 mg arm and 1% in the placebo arm (*P* < 0.0001) (Fig. 3) [34]. Responses were durable; median duration of spleen volume response in the fedratinib 400 mg arm was 18.2 months [34]. In subgroup analyses, achievement of spleen responses with fedratinib was not appreciably affected by *JAK2* mutational status, MF disease subtype, disease risk status, or baseline platelet count greater than or less than 100 × 10^9^/L [24]. No meaningful changes were detected in *JAK2*V617F allele burden in any treatment arm at 24 weeks.

**Fig. 4:**
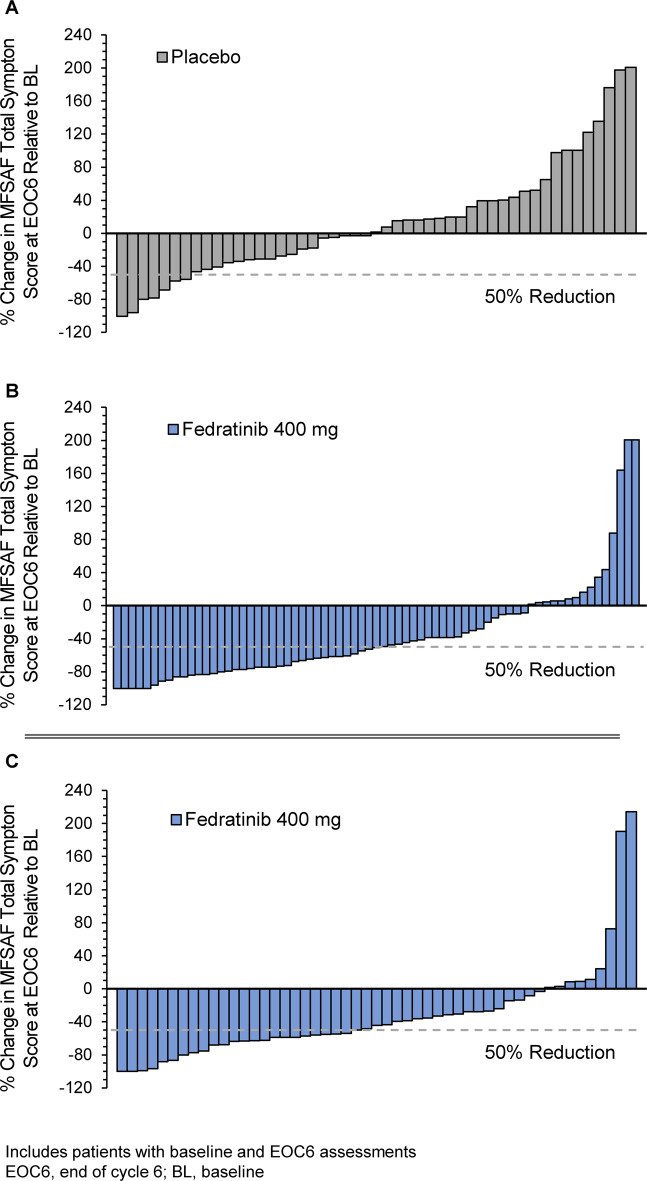
Symptom response. Individual changes in MFSAF total symptom score (TSS) at the end of treatment cycle 6 in JAKARTA with (**a**) placebo and (**b**) fedratinib 400 mg/day; [34] and in JAKARTA2 with fedratinib 400 mg/day (**c**) in patients previously treated with ruxolitinib [71].

At EOC6, symptom response rate among evaluable patients with baseline MFSAF TSS scores > 0 (*n* = 89) was 40% in the fedratinib 400 mg arm and 9% in the placebo arm (*n* = 81) (*P* < 0.001) (Fig. 4) [34]. TSS decreased from baseline by week 4 of fedratinib treatment, with continued symptom improvements sustained through week 24 [24].

### JAKARTA2

The open-label, single-arm, phase II JAKARTA2 study assessed the clinical activity of fedratinib at a starting dose of 400 mg/day in patients with intermediate or high-risk primary or secondary MF previously treated with ruxolitinib [28, 71]. The study began in 2011, at around the same time that ruxolitinib was approved by the US FDA for treatment of MF, and clinical experience with ruxolitinib was limited. Per protocol, eligible patients were required to be ruxolitinib-resistant following at least 14 days of prior ruxolitinib therapy, or ruxolitinib-intolerant after any duration of prior ruxolitinib treatment. Ruxolitinib resistance or intolerance was determined at the discretion of the treating investigator. The primary endpoint was SVRR, and symptom response rate in the MFSAF Analysis Population (i.e., patients with evaluable TSS data at baseline and at ≥1 post-baseline assessment) was the key secondary endpoint.

In all, 97 patients enrolled and received at least 1 fedratinib dose and comprised the ITT Population. Patients in JAKARTA2 receiving fedratinib second-line appeared to have more advanced disease than JAK-inhibitor-naïve patients who enrolled in JAKARTA. For example, one-third of patients began JAKARTA2 with platelet counts <100 × 10^9^/L (vs. 15% of patients in JAKARTA) and more than one-half of all patients (53%) had hemoglobin levels <10 g/dL (vs. 34% of patients in the fedratinib 400 mg arm in JAKARTA) (Supplementary Table 1) [24, 28]. Median spleen volume at baseline was 2894 mL (range 737–7815) and median spleen size was 18.0 cm (5.0–36.0).

In the original JAKARTA2 analysis [28], fedratinib was associated with a 55% SVRR and 26% symptom response rate in the “Per-protocol Population,” comprising 83 patients who had a baseline and ≥1 post-baseline spleen volume measurement and who had no important protocol deviations that could impact efficacy. This original analysis employed a prespecified last-observation-carried-forward statistical method that allowed patients with missing data at EOC6 to be eligible for response, based on their last post-baseline spleen volume assessment [28].

Since experience with ruxolitinib has grown over the years following the initiation of JAKARTA2, an updated rigorous analysis of JAKARTA2 data was recently conducted employing ITT analysis principles for all treated patients (ITT Population), and for a subset of patients who met new, more stringent definitions of ruxolitinib relapsed, refractory, or intolerant (Stringent Criteria Cohort) than were used in the initial analysis (Table 2). Moreover, a sensitivity analysis was performed in patients who met the new stringent criteria for ruxolitinib failure and who reached treatment cycle 6 or discontinued from the study prior to cycle 6 for reasons other than study termination (Sensitivity Analysis Cohort); the primary endpoint would have been least affected by early termination of the study in this subgroup.

**Table 2:** JAKARTA2 analysis populations [71].

ITT population (*N* = 97)	Stringent criteria cohort (*n* = 79)	Sensitivity analysis cohort (*n* = 66)
Ruxolitinib resistant or intolerant to ruxolitinib per investigator discretion: – Resistant: No response, stable disease, evidence of disease progression, or loss of response to ruxolitinib for ≥14 days – Intolerant: Discontinuation due to unacceptable toxicity after any duration of RUX exposure	Relapsed: Ruxolitinib treatment for ≥3 months with spleen regrowth, defined as <10% SVR or <30% decrease in spleen size from baseline, following an initial response. Response to ruxolitinib is defined as a ≥35% reduction in spleen volume from baseline, or a ≥50% reduction in spleen size for baseline spleen >10 cm; a non-palpable spleen for baseline spleen size between 5 and 10 cm; or not eligible for spleen response for baseline spleen <5 cm	Subgroup of patients within the Stringent Criteria Cohort who received 6 cycles of fedratinib therapy or discontinued before cycle 6 for reasons other than “study terminated by sponsor”
	Refractory: Ruxolitinib treatment for ≥3 months with <10% SVR or <30% decrease in spleen size from baseline	
	Intolerant: Ruxolitinib treatment for ≥28 days complicated by development of RBC transfusion requirement (≥2 units per month for 2 months); or grade ≥ 3 thrombocytopenia, anemia, hematoma and/or hemorrhage while receiving ruxolitinib	

*RBC* red blood cell, *SVR* spleen volume reduction.

The Stringent Criteria Cohort included 79 patients; 18 patients were excluded because they had an adequate response to ruxolitinib, were missing ruxolitinib response data, or had received <3 months of ruxolitinib. A total of 66 patients (68%) who met the stringent criteria for ruxolitinib failure, and had the opportunity to receive at least 6 cycles of fedratinib, comprised the Sensitivity Analysis Cohort. Baseline characteristics in the Stringent Criteria Cohort and Sensitivity Analysis Cohort showed no overt differences from those of the ITT Population.

On average, patients in JAKARTA2 had received substantial prior treatment with ruxolitinib before study entry: median prior exposure to ruxolitinib in the ITT Population was 10.7 months (range 0.1–62.4) and median cumulative prior ruxolitinib dose was 9540 mg (80–50,480). In both the Stringent Criteria and Sensitivity Analysis Cohorts, median prior ruxolitinib treatment duration was 11.5 months (range 1.0–62.4). In the Stringent Criteria Cohort, 18 patients (23%) had relapsed after achieving initial response with ruxolitinib, 47 patients (59%) were ruxolitinib-refractory, and 14 patients (18%) were ruxolitinib-intolerant; median prior ruxolitinib exposures in these subgroups were 11.8, 11.4, and 8.7 months, respectively.

The median number of fedratinib cycles received in the ITT Population at the time of the clinical hold was 6 (range 1–20) [71]. In the updated analysis, SVRR at EOC6 in the ITT Population was 31% (95% CI 22%, 41%) at EOC6 (Fig. 3). The Kaplan–Meier estimated duration of spleen volume response was not estimable (NE); among 47 patients who attained a spleen volume response at any time during fedratinib treatment, only 25% had a duration of response of <9.4 months (95% CI 7.2, NE). Response rates in the Stringent Criteria and Sensitivity Analysis cohorts supported findings in the ITT Population: SVRRs in these groups were 30% (95% CI 21%, 42%) and 36% (25%, 49%), respectively. In subgroup analyses, SVRRs were not significantly influenced by reason for prior ruxolitinib failure (relapsed/refractory or intolerant), baseline platelet count greater than or less than 100 × 10^9^/L, or baseline hemoglobin level greater than or less than 10 g/dL (Supplementary Table 2).

In the MFSAF Analysis Population (*N* = 90), the symptom response rate at EOC6 was 27% (95% CI 18%, 37%). Among 51 patients with evaluable TSS data at both baseline and EOC6, 82% reported improvement in symptom severity at EOC6 (Fig. 4). Symptom response rates in the Stringent Criteria Cohort (*n* = 74) and in the Sensitivity Analysis Cohort (*n* = 62) again supported results in the ITT Population (27% and 32%, respectively).

## Safety

Fedratinib safety has been assessed in 608 patients treated with multiple doses of fedratinib in clinical trials, including 459 patients with MF [34]. Among all 608 patients, median treatment exposure was 37 weeks and the median number of cycles initiated was 9. In all, 59% of the 608 patients were exposed to fedratinib for 6 months or longer and 39% were exposed for 12 months or longer [34]. The most common TEAEs in these patients were diarrhea, nausea, anemia, vomiting, fatigue, thrombocytopenia, and constipation. Gastrointestinal TEAEs tended to occur during early treatment and decreased in frequency as treatment continued [24, 34, 35]. No unexpected safety signals have emerged in fedratinib clinical trials in patients who received more than 6 cycles of fedratinib treatment [35, 58].

### JAKARTA

In the randomized portion of the placebo-controlled, phase III JAKARTA study, 79/96 patients (82%) in the fedratinib 400 mg/day arm and 62/95 patients (65%) in the placebo arm completed 6 treatment cycles [24]. Overall, proportions of patients reported to have had any TEAE were 100% in the fedratinib 400 mg arm, and 94% in the placebo arm [24]. Serious TEAEs occurred in 21% of patients in the fedratinib 400 mg arm [34], and 23% in the placebo arm [24, 34]. TEAEs leading to fedratinib dose reductions and interruptions in the 400 mg/day arm occurred in 19% and 21% of patients, respectively, and to permanent treatment discontinuation in 14% of patients [34].

The most common TEAEs during the 24-week randomized treatment phase in the fedratinib 400 mg arm were diarrhea, nausea, and anemia; and in the placebo arm were diarrhea, fatigue/asthenia, and nausea (Table 3) [34]. The median time to onset of any grade nausea, vomiting, and diarrhea was 1 day, with 75% of cases occurring within 2 weeks of initiating treatment [34].

**Table 3:** JAKARTA: adverse events reported in ≥5% patients receiving fedratinib 400 mg, with a difference of >5% between the fedratinib 400 mg/day and placebo arms; and selected laboratory abnormalities that worsened from baseline (≥20% of patients), with a difference of >10% between fedratinib 400 mg/day and placebo during randomized treatment [34].

	INREBIC 400 mg (*n* = 96)		Placebo (*n* = 95)	
	All grades %	Grade ≥ 3^a^ %	All grades %	Grade ≥ 3 %
Adverse reaction^b^				
Diarrhea	66	5	16	0
Nausea	62	0	15	0
Anemia	40	30	14	7
Vomiting	39	3.1	5	0
Fatigue or asthenia	19	5	16	1.1
Muscle spasms	12	0	1.1	0
Blood creatinine increased	10	1	1.1	0
Pain in extremity	10	0	4.2	0
Alanine aminotransferase increased	9	0	1.1	0
Headache	9	0	1.1	0
Weight increased	9	0	4.2	0
Dizziness	8	0	3.2	0
Bone pain	8	0	2.1	0
Urinary tract infection^c^	6	0	1.1	0
Dysuria	6	0	0	0
Aspartate aminotransferase increased	5	0	1.1	0
Selected laboratory abnormalities				
Hematology				
Anemia	74	34	32	10
Thrombocytopenia	47	12	26	10
Neutropenia	23	5	13	3.3
Biochemistry				
Creatinine increased	59	3.1	19	1.1
ALT increased	43	1	14	0
AST increased	40	0	16	1.1
Lipase increased	35	10	7	2.2
Hyponatremia	26	5	11	4.3
Amylase increased	24	2.1	5	0

^a^Only 1 Grade 4 event (anemia).

^b^CTCAE version 4.03.

^c^Includes cystitis.

Laboratory-assessed anemia and thrombocytopenia occurred at higher rates with fedratinib 400 mg than with placebo (Table 3) [34]. New or worsening grade 3 anemia occurred in 34% of patients treated with fedratinib 400 mg, with median time to onset of ~2 months, and 75% of cases occurring within 3 months. Mean hemoglobin levels reached nadir after 12–16 weeks with partial recovery and stabilization after 16 weeks [34]. Permanent discontinuation of fedratinib 400 mg due to anemia occurred in 1% of patients. New or worsening grade ≥ 3 thrombocytopenia during the randomized treatment period occurred in 12% of fedratinib-treated patients, with median time to onset of ~1 month and 75% of cases occurring within 4 months. Permanent discontinuation of treatment due to thrombocytopenia and bleeding that required clinical intervention both occurred in 2.1% of INREBIC-treated patients [34].

### JAKARTA2

In the phase II, single-arm JAKARTA2 study in patients previously treated with ruxolitinib, 33% of patients began the study with platelet counts < 100 × 10^9^/L and 53% had baseline hemoglobin concentrations <10 g/dL. Grades 3–4 anemia and thrombocytopenia were more commonly reported in patients with low baseline platelet counts: 46% and 49% of patients with platelet counts < 100 × 10^9^/L experienced grades 3–4 anemia and thrombocytopenia, respectively, vs. 34% and 8% of patients with platelet counts ≥ 100 × 10^9^/L. Supplementary Table 3 shows the most common TEAEs and selected laboratory abnormalities. Treatment interruptions of ≥7 days occurred in 26% of patients and fedratinib dose-reduction occurred in 26%. The most common TEAEs requiring dose-interruption or dose-reduction were nausea (8%), anemia (8%), diarrhea (7%), and thrombocytopenia (6%). Nineteen patients (20%) permanently discontinued fedratinib due to a TEAE; diarrhea and thrombocytopenia (*n* = 2 each) were the only TEAEs leading to discontinuation in >1 patient. Fedratinib-related TEAEs led to permanent treatment discontinuation for 10 patients (10%). Two patients discontinued fedratinib due to treatment-related anemia or thrombocytopenia (*n* = 1 each). One case of grade 3 hepatic encephalopathy occurred in the JAKARTA2 study, and no confirmed case of Wernicke’s encephalopathy (WE) occurred.

### Encephalopathy

The fedratinib development program was placed on clinical hold in November 2013 due to suspected cases of drug-related WE, a rare neurological disorder induced by a deficiency of thiamine (vitamin B1). Among the more than 600 patients treated with multiple fedratinib doses in clinical trials, 8 (1.3%) potential cases of WE were reported and 1 case (0.16%) was fatal [72]. Most events resolved with some residual neurological symptoms. Retrospective analysis of potential WE events suggested that affected patients experienced predisposing conditions known to lead to WE in any population (e.g., underlying malnutrition and uncontrolled gastrointestinal events) [72, 73]. The clinical hold was lifted in August 2017 after additional safety information was provided to the FDA. The fedratinib prescribing information includes a “black box” warning of the potential for encephalopathy, including WE, during fedratinib treatment. Patients should have normal thiamine levels before starting fedratinib, and thiamine monitoring periodically during treatment as clinically indicated is recommended [34].

## Discussion

The recent approval of fedratinib provides a much-needed addition to the limited therapeutic options available for transplant-ineligible patients with intermediate- or high-risk MF. In both JAK-inhibitor-naïve patients and those previously treated with ruxolitinib, fedratinib induced spleen reductions and improved MF symptom burden.

Many patients with MF have benefited from spleen size reductions and symptom improvements with ruxolitinib therapy [74–77]. However, as many as one-half of ruxolitinib-treated patients require dose reductions or interruptions [75, 78, 79], often because of cytopenias, and recommended dosing for patients with pretreatment platelet counts < 100 × 10^9^/L (5 mg BID [29]) may induce suboptimal responses [80]. Moreover, a majority of patients discontinue ruxolitinib within 3–5 years [74–77]. Prognosis for patients with MF who discontinue ruxolitinib is poor, with median survival ranging from 6 to 14 months [32, 81, 82]. Fedratinib may fulfill an important unmet need for patients who have discontinued ruxolitinib due to resistance or intolerance.

Treatment with fedratinib in JAK-inhibitor-naïve patients showed robust activity for improving splenomegaly and symptom burden [24–26]. Moreover, fedratinib showed more potent therapeutic activity when used in patients previously exposed to ruxolitinib than other JAK inhibitors in later stages of clinical development (Table 4). For patients in JAKARTA2 who met updated, stringent criteria for ruxolitinib failure, 30% achieved a spleen volume response with fedratinib, whereas the SVRR in patients previously exposed to ruxolitinib who received the JAK2/FLT3 inhibitor, pacritinib, in the PERSIST-2 trial was 10%, and the SVRR in a similar patient population treated with momelotinib, a dual JAK1/JAK2 inhibitor, in the SIMPLIFY-2 trial was 7% [28, 83, 84].

**Table 4:** Spleen volume response rates and symptom response rates following 24 weeks of treatment with JAK inhibitors in late-stage clinical development.

	First-line MF treatment					Prior ruxolitinib exposure		
	JAKARTA [34] fedratinib 400 mg QD *N* = 96	COMFORT-I [26] ruxolitinib 15–20 mg BID *N* = 155	COMFORT-II [25] ruxolitinib 15–20 mg BID *N* = 146	PERSIST-1 [27] pacritinib 400 mg QD *N* = 220	SIMPLIFY-1 [78] momelotinib 200 mg QD *N* = 214	JAKARTA2 [71] fedratinib 400 mg QD *N* = 97	PERSIST-2 [83] pacritinib 400 mg *N* = 210	SYMPLIFY-2 [84] momelotinib 200 mg QD *N* = 104
Primary kinases inhibited	JAK2/FLT3	JAK1/JAK2	JAK1/JAK2	JAK2/FLT3	JAK1/JAK2	JAK2/FLT3	JAK2/FLT3	JAK1/JAK2
Spleen volume response rate^a^	37%^b^	42%	32%	19%	27%	31%	10%^c^	7%
Symptom response rate^d^	40%	46%	N/A^e^	25%^f^	28%	27%	21%^c^	26%

*EORTC* QLQ-C30 European Organisation for Research and Treatment of Cancer Quality-of-Life Questionnaire, *MF* myelofibrosis, *MFSAF* MF Symptom Assessment Form, *MPN-SAF* Myeloproliferative Neoplasm Symptom Assessment Form.

^a^Proportion of patients who achieved a ≥35% decrease in spleen volume from baseline through week 24.

^b^Response rate at 24 weeks, confirmed 4 weeks later [34]. Unconfirmed response rate at 24 weeks was 47% [24].

^c^Among 62 patients in PERSIST-2 who had previously been treated with ruxolitinib.

^d^Proportion of patients who experienced a ≥50% decrease in total symptom score per the MFSAF or the MPN-SAF, from baseline through week 24.

^e^Symptom changes in COMFORT-II were assessed according to the EORTC QLQ-C30 questionnaire [25].

^f^Composite symptom response rate using both the MPN-SAF (original version) and MPN-SAF version 2.0 questionnaires [27].

Preclinical data established the selectivity and potency of JAK2 inhibition by fedratinib [37]. In addition to its role in cell proliferation, aberrant JAK2 signaling leads to several changes including increased NF-κB signaling via chromatin changes that contribute to MPN-associated inflammation [47]. In an MPN animal model, JQ1, a potent BET inhibitor, and ruxolitinib, each given alone, attenuated NF-κB activation and reduced inflammatory cytokine production. However, combining JQ1 and ruxolitinib produced synergistic therapeutic effects, leading to substantial reductions in plasma levels of inflammatory cytokines, reduced disease burden, and reversed bone marrow fibrosis in vivo [47]. Unlike ruxolitinib, which has not exhibited inhibitory activity against bromodomains, fedratinib has potent dual activity against JAK2 and BRD4 [45], and fedratinib monotherapy has been shown to reduce inflammatory cytokine levels in patients with MF [55] and may ameliorate bone marrow fibrosis [68]. Combination therapy with the specific BET inhibitor, CPI-0610, and ruxolitinib, and CPI-0610 monotherapy are currently under evaluation in a phase II, open-label clinical trial in patients with MF who are JAK-inhibitor naïve or refractory or intolerant to prior ruxolitinib treatment [85, 86]. Observed cytokine regulation during fedratinib treatment, including downregulation of pro-inflammatory TNF-α, may contribute to improvements in MF symptoms, and upregulation of anti-inflammatory and anti-fibrotic adiponectin may affect bone marrow fibrosis [55]. The effect of fedratinib on *JAK2*V617F allele burden is uncertain, with some data suggesting activity against the malignant clone and other findings suggesting no noticeable effects [24, 35, 55, 58]. These findings warrant further study in larger patient populations.

Because the JAK/STAT signaling pathway is implicated in cancer cell development and survival, and in chronic inflammation, fedratinib may also have utility in other diseases [87, 88]. As mentioned, fedratinib-induced inhibition of activated hepatic stellate cells and inflammatory macrophage infiltration in vivo suggests a potential therapeutic role for fedratinib in hepatic fibrosis [69]. Dysregulated JAK/STAT signaling may be involved in resistance mechanisms against molecularly targeted drugs [87]. In vitro, fedratinib significantly enhanced the cytotoxicity of erlotinib in erlotinib-resistant non-small cell lung carcinoma (NSCLC) cells with epidermal growth factor receptor mutations, and inhibited tumor growth of erlotinib-resistant NSCLC cells in vivo [89].

Effective management of fedratinib-related TEAEs can facilitate long-term therapy. Hematologic events are anticipated with JAK2 inhibitors due to their mechanism of action [90]. These events typically occur within the first 3–4 months of initiating fedratinib therapy and hematology counts tend to improve over time during treatment [34, 35, 55]. Grades 3–4 laboratory abnormalities of anemia and thrombocytopenia were more frequent in the JAKARTA2 study in patients previously treated with ruxolitinib than in the JAKARTA trial, as might be expected for patients with more advanced disease and who were more likely to have low platelet counts and hemoglobin concentrations at study entry [24]. In both studies, permanent discontinuation of fedratinib due to these events was infrequent (2–3%), suggesting that these events could be managed effectively while continuing fedratinib treatment. Hematologic TEAEs can be managed with supportive interventions, including transfusions, and fedratinib dose modifications may be needed if supportive interventions are insufficient. Although not contraindicated, the benefit/risk ratio of fedratinib therapy should be carefully measured in patients with highly transfusion-dependent anemia or profound thrombocytopenia.

Off-target inhibition of FLT3 may be implicated in the occurrence of gastrointestinal events [91], which are the most common TEAEs seen with fedratinib. These events, the majority of which are grades 1–2 severity, occur mainly during early treatment and decrease over time [24, 34], so appropriate patient education and expectation setting is important to ensure drug adherence. Gastrointestinal events can be managed with prophylaxis for nausea and vomiting (e.g., with a 5-HT3 receptor antagonist) or prompt treatment of diarrhea at first onset of symptoms. Though food does not meaningfully alter the bioavailability of fedratinib, taking fedratinib with a high-fat meal may help reduce nausea and vomiting [54].

Patients with MF may have more risk factors for developing Wernicke’s encephalopathy than persons without MF, regardless of treatment [92]. Nevertheless, Wernicke’s encephalopathy can be caused by thiamine deficiency secondary to persistent vomiting, especially in an already malnourished individual [73, 93]. Preclinical studies of fedratinib at clinically relevant concentrations have shown fedratinib does not have an effect on the thiamine receptor function in the gastrointestinal tract or the brain [94, 95]. However, because gastrointestinal events are common, proactive management of nausea, vomiting, and diarrhea may help prevent thiamine deficiency during fedratinib treatment and risk-mitigation strategies for Wernicke’s encephalopathy, including routine monitoring of thiamine as appropriate, are recommended for patients with MF receiving fedratinib [34].

Because of the clinical hold, long-term data for fedratinib are limited. Two phase III clinical trials, FREEDOM (NCT03755518) and FREEDOM2 (NCT03952039), are currently underway to evaluate the long-term safety, efficacy, and effect on overall survival of fedratinib 400 mg/day in patients with intermediate-2 or high-risk MF previously treated with ruxolitinib. FREEDOM is a single-arm trial and FREEDOM2 is a randomized trial that compares fedratinib with best available therapy (BAT). Both studies employ risk-mitigation strategies for Wernicke’s encephalopathy and proactive management of gastrointestinal events to prevent thiamine deficiency.

Fedratinib is a promising new therapy for patients with advanced MF who until recently have had limited treatment options. More extensive clinical experience with fedratinib is needed to determine the duration of spleen response during treatment, the potential to develop secondary resistance, and whether biomarkers may identify a subset of patients with MF most likely to respond to the drug.

## Supplementary information

Supplementary File
