# Supplementary material for: Fedratinib, a newly approved treatment for patients with myeloproliferative neoplasm-associated myelofibrosis
Source: Leukemia. 2020 Jul 9;35(1):1–17. doi: 10.1038/s41375-020-0954-2 (PMC7787977; doi:10.1038/s41375-020-0954-2)
Supplement: Supplementary file 1 — Supplementary File [file 41375_2020_954_MOESM1_ESM.pdf]

Supplementary Table 1. Baseline characteristics among patients enrolled in JAKARTA or JAKARTA2[34,71]

	JAKARTA[34]		JAKARTA2[71]
	**Placebo (N=96)**	**Fedratinib 400 mg/day (N=96)**	**Fedratinib 400 mg/day (N=97)**
Age, years, median (range)	66 (27-85)	63 (39-86)	67 (38-83)
Male sex, n (%)	55 (57)	54 (56)	53 (55)
MF subtype, n (%)			
Primary	58 (60)	62 (65)	53 (55)
Post-PV	27 (28)	24 (25)	25 (26)
Post-ET	11 (12)	10 (10)	19 (20)
IPSS risk status, n (%)			
Intermediate	46 (48)	57 (59)	63 (65)
High	50 (52)	39 (41)	34 (35)
Platelet count			
Median (range)	187 (52-1075)	221 (31-1155)	NR
50 to < 100 × 10^9/L, n (%)	18 (19)	14 (15)	33 (34)
≥100 × 10^9/L, n (%)	77 (80)	82 (85)	64 (66)
Hemoglobin			
Median, g/dL (range)	10.1 (4.5-17.1)	10.7 (4.8-16.8)	NR
< 10 g/dL, n (%)	NR	NR	51 (53)
≥ 10 g/dL, n (%)	NR	NR	46 (47)
Spleen volume, cm^3, median (range)	2660 (662–7911)	2652 (316–6430)	2894 (737–7815)
Palpable spleen size, cm, median (range)	17 (5–40)	16 (5–40)	18 (5–36)
JAK2 mutation status, n (%)			
Mutant	59 (62)	62 (65)	61 (63)
Wild-type	32 (33)	30 (31)	29 (30)
Missing	5 (5)	4 (4)	7 (7)

ECOG, Eastern Cooperative Oncology Group; IPSS, International Prognostic Scoring System; MF, myelofibrosis; NR, not reported; post-ET, post-essential thrombocythemia; post-PV, post-polycythemia vera.

Supplementary Table 2. Spleen volume response rates with fedratinib 400 mg at the end of treatment cycle 6 in the JAKARTA2 study, by outcome of prior ruxolitinib therapy, baseline platelet count, and baseline hemoglobin concentration[71]

	ITT Population N=97		Stringent Criteria Cohort n=79		Sensitivity Analysis Cohort n=66	
**Prior ruxolitinib outcome**	**Resistant* n=64**	**Intolerant* n=32**	**Relapsed/ refractory† n=65**	**Intolerant† n=14**	**Relapsed/ refractory† n=56**	**Intolerant† n=10**
**n (%) [95% CI]**	21 (33) [22, 46]	9 (28) [14, 47]	20 (31) [20, 43]	4 (29) [8, 58]	20 (36) [23, 50]	4 (40) [12, 74]
**Baseline platelet count**	**<100 × 10^9/L n=33**	**≥100 × 10^9/L n=64**	**<100 × 10^9/L n=28**	**≥100 × 10^9/L n=51**	**<100 × 10^9/L n=26**	**≥100 × 10^9/L n=40**
**n (%) [95% CI]**	12 (36) [20, 55]	18 (28) [18, 41]	11 (39) [22, 59]	13 (26) [14, 40]	11 (42) [23, 63]	13 (33) [19, 49]
**Baseline hemoglobin concentration**	**<10 g/dL n=51**	**≥10 g/dL n=46**	**<10 g/dL n=46**	**≥10 g/dL n=33**	**<10 g/dL n=40**	**≥10 g/dL n=26**
**n (%) [95% CI]**	14 (28) [16, 42]	16 (35) [21, 50]	12 (26) [14, 41]	12 (36) [20, 55]	12 (30) [17, 47]	12 (46) [27, 67]

*Per enrolling investigator. One patient was classified as "Other: lack of efficacy".
†Relapsed/refractory or intolerant per updated stringent criteria.

Supplementary Table 3. JAKARTA2: treatment-emergent adverse events with fedratinib 400 mg reported in >10% of patients in the ITT Population, and hematology and biochemistry laboratory abnormalities of interest[71]

	ITT Population (N=97)		Stringent Criteria Cohort (n=79)		Sensitivity Analysis Cohort (n=66)	
	Any Grade n (%)	Grade 3–4 n (%)	Any Grade n (%)	Grade 3–4 n (%)	Any Grade n (%)	Grade 3–4 n (%)
**Preferred term**						
Diarrhea	60 (62)	4 (4)	51 (65)	3 (4)	44 (67)	3 (5)
Nausea	54 (56)	0	42 (53)	0	35 (53)	0
Anemia	47 (49)	37 (38)	44 (56)	35 (44)	39 (59)	31 (47)
Thrombocytopenia	26 (27)	21 (22)	21 (27)	16 (20)	20 (30)	15 (23)
Vomiting	40 (41)	0	35 (44)	0	31 (47)	0
Constipation	20 (21)	1 (1)	17 (22)	0	15 (23)	0
Pruritus	17 (18)	0	14 (18)	0	12 (18)	0
Fatigue	15 (16)	2 (2)	11 (14)	1 (1)	10 (15)	1 (2)
Cough	13 (13)	0	12 (15)	0	9 (14)	0
Headache	13 (13)	1 (1)	10 (13)	1 (1)	9 (14)	1 (2)
Urinary tract infection	12 (12)	0	11 (14)	0	11 (17)	0
Abdominal pain	12 (12)	2 (2)	9 (11)	1 (1)	8 (12)	1 (2)
Dyspnea	12 (12)	1 (1)	9 (11)	1 (1)	8 (12)	1 (2)
Asthenia	11 (11)	1(1)	10 (13)	1 (1)	7 (11)	1 (2)
Dizziness	11 (11)	0	9 (11)	0	7 (11)	0
Pyrexia	11 (11)	1 (1)	7 (9)	0	7 (11)	0
**Hematology,***						
Anemia	96 (99)	45 (46)	79 (100)	43 (54)	66 (100)	37 (56)
Thrombocytopenia	68 (70)	23 (24)	58 (73)	18 (23)	51 (77)	16 (24)
Neutrophil count decrease	23 (24)	7 (7)	22 (28)	7 (9)	20 (30)	7 (11)
**Biochemistry,***						
Creatinine increased	72 (74)	0	57 (72)	0	48 (73)	0
AST increased	46 (47)	1 (1)	39 (49)	1 (1)	33 (50)	1 (2)
ALT increased	44 (45)	2 (2)	41 (52)	2 (3)	35 (53)	2 (3)
Lipase increased	25 (26)	8 (8)	20 (25)	6 (8)	17 (26)	5 (8)
Amylase increased	17 (18)	3 (3)	15 (19)	2 (3)	10 (15)	2 (3)

TEAEs were classified according to the Medical Dictionary for Regulatory Activities (MedDRA) version 20.1, and graded according to National Cancer Institute Common Terminology Criteria for Adverse Events (NCI-CTCAE) version 4.03.
NR, not reported; TEAE, treatment-emergent adverse event.
*Hematologic and biochemical events (worst grade, including baseline) were assessed by laboratory analysis.
ALT, alanine aminotransferase; AST, aspartate aminotransferase
